# ForgIng New paths in DIabetes PrevenTion (FINDIT): Study Protocol for a Randomized Controlled Trial

**DOI:** 10.1186/s13063-017-1887-6

**Published:** 2017-04-08

**Authors:** Jeffrey T. Kullgren, Bradley Youles, Shaina Shetty, Caroline Richardson, Angela Fagerlin, Michele Heisler

**Affiliations:** 1 VA Center for Clinical Management Research, VA Ann Arbor Healthcare System, PO Box 130170, Ann Arbor, MI 48113-0170 USA; 2 Department of Internal Medicine, University of Michigan Medical School, Ann Arbor, MI USA; 3 University of Michigan Institute for Healthcare Policy and Innovation, Ann Arbor, MI USA; 4 Department of Family Medicine, University of Michigan Medical School, Ann Arbor, MI USA; 5 Salt Lake City VA Medical Center, Salt Lake City, UT USA; 6 Department of Population Health Studies, University of Utah, Salt Lake City, UT USA; 7 Department of Health Behavior and Health Education, University of Michigan, Ann Arbor, MI USA

**Keywords:** Diabetes prevention, Patient engagement, Screening, Patient education, Brief intervention

## Abstract

**Background:**

Prediabetes is an asymptomatic condition in which patients’ blood glucose levels are higher than normal but do not meet diagnostic criteria for type 2 diabetes mellitus (T2DM). A key window of opportunity to increase engagement of patients with prediabetes in strategies to prevent T2DM is when they are screened for T2DM and found to have prediabetes, yet the effects of this screening and brief counseling are unknown.

**Methods:**

In this parallel-design randomized controlled trial we will recruit 315 non-diabetic patients from the Ann Arbor VA Medical Center (AAVA) who have one or major risk factors for T2DM and an upcoming primary care appointment at the AAVA, but have not had a hemoglobin A1c (HbA1c) test to screen for T2DM in the previous 12 months. After informed consent, participants will complete a baseline survey and be randomly assigned to, at the time of their next primary care appointment, one of two arms: (1) to have a hemoglobin A1c (HbA1c) test to screen for T2DM and receive brief, standardized counseling about these results or (2) to review a brochure about clinical preventive services. Participants will complete surveys 2 weeks, 3 months, and 12 months after their primary care appointment, and a weight measurement 12 months after their primary care appointment. The primary outcome is weight change after 12 months. The secondary outcomes are changes in perception of risk for T2DM; knowledge of T2DM prevention; self-efficacy and motivation to prevent T2DM; use of pharmacotherapy for T2DM prevention; physical activity; participation in weight management programs; and mental health. Quantitative analyses will compare outcomes among participants in the HbA1c test arm found to have prediabetes with participants in the brochure arm. Among participants in the HbA1c test arm found to have prediabetes we will conduct semi-structured interviews about their understanding of and reactions to receiving a prediabetes diagnosis.

**Discussion:**

This trial will generate foundational data on the effects of a prediabetes diagnosis and brief counseling on patients’ preventive behaviors and mediators of these behaviors that will enable the development of novel strategies to improve patient engagement in T2DM prevention.

**Trial registration:**

ClinicalTrials.gov, NCT02747108. Registered on 18 April 2016.

**Electronic supplementary material:**

The online version of this article (doi:10.1186/s13063-017-1887-6) contains supplementary material, which is available to authorized users.

## Background

Prediabetes is an asymptomatic condition in which patients’ blood glucose levels are higher than normal but not high enough to meet diagnostic criteria for type 2 diabetes mellitus (T2DM). The American Diabetes Association (ADA) defines prediabetes as either fasting plasma glucose of 100 to 125 mg/dL, 2-hour plasma glucose of 140 to 199 mg/dL after a 75-g oral glucose tolerance test, or hemoglobin A1c (HbA1c) of 5.7 to 6.4% [1]. Currently 38% of adults in the USA have prediabetes [2], which is associated with an approximately threefold greater annual incidence of T2DM [3] and an approximately 50% greater risk of cardiovascular disease [4–6].

Fortunately, patients with prediabetes can significantly reduce their risk of developing T2DM through weight loss, physical activity, or pharmacotherapy [7, 8]. The landmark Diabetes Prevention Program (DPP) trial demonstrated that a lifestyle-modification program with the goals of at least 7% weight loss and at least 150 minutes of physical activity per week led to a 58% reduction in the 3-year incidence of T2DM [9]. This was significantly greater than the 31% reduction in the incidence of T2DM that was observed among patients who received metformin 850 mg by mouth twice daily, but progression to T2DM with metformin was still significantly less than the rate of progression in the control group. Due to the effectiveness [8] and cost-effectiveness [10] of these interventions, a number of studies have translated the DPP for use in different populations [11]. Moreover, the National DPP, an initiative led by the US Centers for Disease Control and Prevention, is currently working to disseminate and implement the DPP in communities across the USA [12].

### Importance of patient engagement in diabetes prevention

While there are defined ways to identify patients with prediabetes and evidence-based strategies to prevent or delay their progression to T2DM, the real-world execution of these strategies relies heavily on patient engagement. Specifically, in order for patients with prediabetes to engage in interventions to prevent or delay T2DM they must: (1) believe they have an elevated but modifiable risk of T2DM; (2) be motivated to prevent T2DM; (3) understand strategies they could use to prevent T2DM and be motivated to engage in these strategies; and (4) have the self-efficacy and support needed to sustain these behaviors. These goals are often difficult for at-risk patients to achieve, as evidenced by widespread lack of awareness of a prediabetes diagnosis, [13] frequent underestimation of risk of T2DM [14], and low rates of engagement in behaviors to prevent T2DM [15–17].

### Screening for diabetes as a window of opportunity for diabetes prevention

**Fig. 1:**
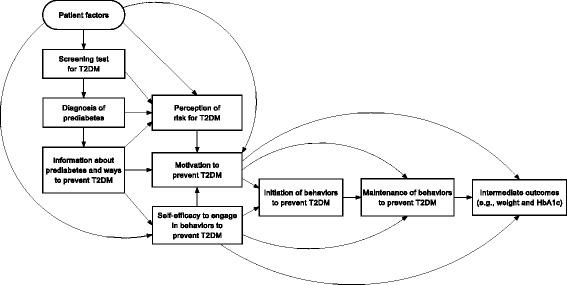
Conceptual model of patient engagement in behaviors recommended for the prevention of type 2 diabetes mellitus (*T2DM*). *HbA1c* hemoglobin A1c

A key opportunity to increase patients’ engagement in evidence-based strategies to prevent T2DM is when they are informed at a primary care appointment that they have prediabetes. This process is depicted in the conceptual model shown in Fig. 1, which incorporates key domains from relevant health behavior theories such as the health belief model [18] and the transtheoretical model [19]. This process of informing patients that they have prediabetes usually starts with a screening laboratory test for T2DM. Patients whose laboratory test results are in the prediabetes range should then be informed by their provider that they have prediabetes and educated about how they can prevent or delay the onset of T2DM by: (1) losing weight and increasing physical activity, (2) participating in an evidence-based weight management program like the DPP; or (3) starting metformin [20]. At this critical stage, patients then should decide whether to engage in any behavior to reduce their risk for T2DM and, if so, which strategy or strategies to pursue.

Qualitative research from the UK suggests that this process of informing patients that they have prediabetes represents a highly “teachable moment” during which patients reflect on their risk of developing T2DM and form their motivation to reduce this risk [21, 22]. While this research indicates that the content of information given to patients when they are identified as having prediabetes is important, it also points to ways in which subtle factors may shape patient engagement. For example, the speed with which patients receive their screening test results, the source of those results (e.g., physician or ancillary provider), and the clarity of recommendations on behavior change may each impact the inferences patients draw about their risk of developing T2DM and their subsequent decisions to engage in behaviors to prevent T2DM [21, 22]. Despite the promise of this process of screening for T2DM to increase patients’ perception of their risk of T2DM, their motivation and self-efficacy to prevent T2DM, and their efforts to modify risk factors for T2DM such as excess weight and lack of physical activity, the effects of a prediabetes diagnosis and brief counseling on these factors remain undefined [21, 23–25]. The objectives of this parallel-design randomized controlled trial (RCT) are to determine the effects of a prediabetes diagnosis and brief counseling on weight change; to examine the effects of a prediabetes diagnosis and brief counseling on engagement in behaviors to prevent T2DM and mediators of these behaviors; and to gain deep insights into patients’ understanding of, and reactions to receiving, a prediabetes diagnosis.

## Methods

### Participants and recruitment

We will recruit 315 non-diabetic patients from the Ann Arbor VA Medical Center (AAVA), who have an upcoming primary care appointment at the AAVA and one or more major risk factors for T2DM, but have not had an HbA1c test to screen for T2DM in the previous 12 months. The full inclusion and exclusion criteria are shown in Table 1.

**Table 1:** Inclusion and Exclusion Criteria

*Inclusion Criteria* ^a^	*Exclusion Criteria*
• Receives primary care at the Ann Arbor VA Medical Center	• Age >75 years^a^
• BMI ≥30 kg/m^2^ or • BMI ≥25 kg/m^2^ with any of the following:	• Type 1 diabetes^a,b^ (ICD-9 250 or ICD-10 E10.9)
• Hypertension (ICD-9 401 or ICD-10 I10)	• Type 2 diabetes^a,b^ (ICD-9 250 or ICD-10 E11)
• Hyperlipidemia (ICD-9 272.4 or ICD-10 E78.5)	• Dementia^a^ (ICD-9 290 or ICD-10 F03)
• Hypoalphalipoproteinemia (ICD-9 272.5 or ICD-10 E78.6)	• End-stage renal disease^a,b^ (ICD-9 585.6 or ICD-10 N18.6)
• Coronary artery disease (ICD-9 414.01 or ICD-10 I25.119)	• Severe chronic obstructive pulmonary disease (ICD-9 496 or ICD-10 J44)^a,b^
• Peripheral vascular disease (ICD-9 443.9 or ICD-10 I73.9)	• Receiving chemotherapy^b^
• Hypertriglyceridemia (ICD-9 272.1 or ICD-10 E78.1)	• New York Heart Association Class III or IV congestive heart failure (ICD-9 428 or ICD-10 I50.20 or ICD-10 I50.30)^a,b^
• Past HbA1c of 5.7 - 6.4	• Pregnant or planning to become pregnant in the next year^b^
• Impaired fasting glucose (ICD-9 790.21 or ICD-10 R73.01)	• Stroke or myocardial infarction in the past 6 months^b^
• Impaired glucose tolerance (ICD-9 790.22 or ICD-10 R73.02)	• Cirrhosis (ICD-9 571 or ICD-10 K74.60)^a^
• Polycystic ovary syndrome (ICD-9 256.4 or ICD-10 E28.2)	• HbA1c test in the past 12 months^a^

^a^Data obtained from the VA Corporate Data Warehouse. ^b^Data obtained from a telephone screening survey administered to patients who are preliminarily eligible based on data from the VA Corporate Data Warehouse. *HbA1c* hemoglobin A1c

Patients who meet preliminary eligibility criteria based on AAVA administrative data will be mailed a study information packet that describes the study and provides them with contact information to inquire further or opt out of being contacted about the study. After 10 days of mailing the letter, a Research Assistant who is blinded to the allocation sequence will call these preliminarily eligible patients to elicit their interest in study participation. For patients who are interested in participating, eligibility criteria that could not be assessed through AAVA administrative data will be evaluated through a brief set of additional screening questions. If at the end of this call the patient is eligible to participate, they will be asked to sign and return the informed consent form that had been mailed to them in their initial study information packet.

### Randomization and study arms

After giving informed consent, the 315 enrolled patients will each complete a baseline survey. Then approximately 2 weeks before their primary care appointment, each patient will be randomly assigned to one of two study groups in a 4:1 ratio (252 to the HbA1c test and brief counseling arm and 63 to the clinical preventive brochure arm) using blocked randomization with variable block lengths. The allocation sequence will be determined using computer-generated random numbers and will be concealed in a password-protected file until study groups are assigned. We chose this 4:1 allocation ratio because in our main analyses we will compare patients in the HbA1c test and brief counseling arm, whose HbA1c is in the prediabetes range to patients in the clinical preventive service brochure arm, and we anticipate that 20% of patients in the HbA1c test and brief counseling arm (i.e., 63 of 252 patients) will have HbA1c in the prediabetes range. All arm assignments will be communicated to patients by phone and a mailed letter, after which patients and Research Assistants will no longer be blinded to the arm assignment, but data analysts will continue to be blinded. Throughout the subsequent 12 months of the study patients in both arms will continue to receive usual care through their VA primary care team, which at the AAVA typically includes routine offers of a referral to the VA weight management program (MOVE) for patients who are overweight or obese.

#### HbA1c test and brief counseling arm

The 252 patients who are randomly assigned to the HbA1c test and brief counseling arm will be asked to have an HbA1c test to screen for T2DM at the AAVA outpatient laboratory. We will then interpret these HbA1c results and group them into the following categories based on American Diabetes Association (ADA) [26] and National DPP [12] guidelines:

- HbA1c <5.7% = normal (i.e., neither prediabetes nor T2DM)
- HbA1c ≥5.7% and <6.5% = prediabetes
- HbA1c ≥6.5% = T2DM

These HbA1c results will be immediately communicated to and interpreted for each patient’s VA primary care physician in a brief note in the VA Computerized Patient Record System (CPRS). Within 2 weeks of their primary care appointment, a Research Assistant will provide to the patient standardized brief phone counseling about their HbA1c result (Additional file 1), and will mail to the patient a summary of this standardized brief counseling (Additional file 2). These processes will provide patients and physicians with an opportunity to discuss the HbA1c result and make plans for follow-up information and support, yet will ensure that all patients will receive their HbA1c result within 2 weeks of the test, in accordance with VA standards.

In this standardized brief counseling and mailed information, patients whose HbA1c is in the normal range will simply be informed of this result. Patients whose HbA1c is in the prediabetes range will receive counseling and information based on the VA/Department of Defense guidelines for prevention of T2DM and will emphasize the risk of progression to T2DM and the rationale for preventive strategies, encourage aerobic exercise and a calorie-restricted diet to promote and maintain weight loss, set a goal of achieving and sustaining weight loss of at least 7% of body weight, and note pharmacotherapy as an option for preventing or delaying T2DM. Patients whose HbA1c is in the T2DM range will be told their HbA1c indicates they may have T2DM and will soon be contacted by their primary care team for follow up.

#### Clinical preventive service brochure arm

The 63 patients who are randomized to the clinical preventive service brochure arm will not have a screening HbA1c test ordered through the study. Instead, these patients will receive usual care and be mailed a brochure from the VA National Center for Health Promotion and Disease Prevention with general information about the value of clinical preventive services such as screenings and immunizations. These patients will be asked to review this information around the time of their primary care appointment. The brochure will be summarized for each patient’s VA primary care physician in a brief VA CPRS note. Within 2 weeks of their primary care appointment, a Research Assistant will call the patient to provide standardized brief counseling based on the information in the brochure. In this way, patients in the clinical preventive service brochure arm will spend an approximately equal amount of time receiving and reviewing information related to prevention as patients randomized to the HbA1c test and brief counseling arm and will thus serve as an attention control group.

### Assessments

#### Surveys

**Fig. 2:**
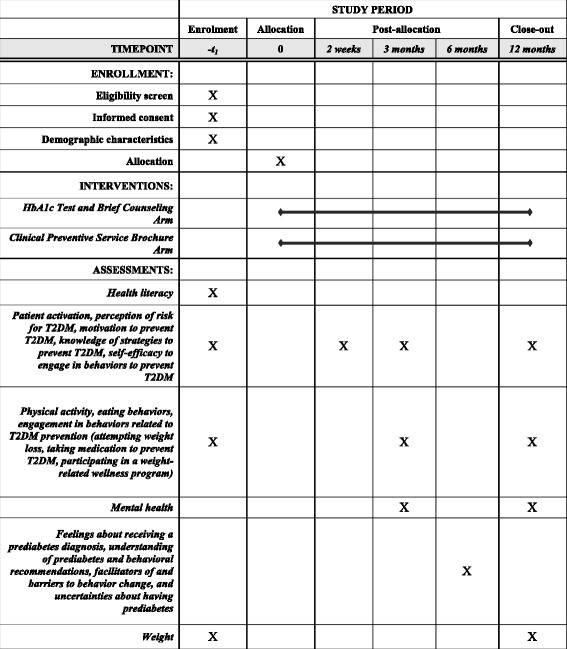
Schedule of enrollment, interventions, and assessments. *T2DM* type 2 diabetes mellitus, *HbA1c* hemoglobin A1c

Patients will complete four surveys: (1) at baseline (i.e., before randomization); (2) within 2 weeks of receiving the HbA1c test results or the clinical preventive service brochure; (3) at 3 months after receiving the HbA1c test results or the clinical preventive service brochure; and (4) at 12 months after receiving the HbA1c test results or the clinical preventive service brochure (Fig. 2).

Patients will be able to complete and submit each survey online (via Qualtrics), by mail, in person, or by telephone. Data from mailed, in person, and telephone surveys will be entered into Qualtrics. The measures contained in each survey are shown in Table 2. For completion of each survey, patients will receive a US$10 gift card. All survey data will be stored in password protected files on a secure server.

**Table 2:** Measures

Category	Source	Measures
Demographic characteristics	VA administrative data at baseline	Age and gender
Demographic characteristics	Baseline survey	Race/ethnicity and education
Health literacy	Baseline survey	Chew health literacy scale
Patient activation	All 4 surveys	Patient activation measure
Perception of risk of T2DM	All 4 surveys	Adriaanse T2DM risk perception scale
Motivation to prevent T2DM	All 4 surveys	Treatment self-regulation questionnaire
Knowledge of strategies to prevent T2DM	All 4 surveys	Knowledge of whether weight loss, physical activity, and pharmacotherapy can prevent T2DM
Self-efficacy to engage in behaviors to prevent T2DM	All 4 surveys	Perceived competence scale for weight loss, increasing physical activity, taking medication
Physical activity	Baseline, 3 month, 12 month surveys	Short form of the international physical activity questionnaire
Eating behaviors	Baseline, 3 month, 12 month surveys	Three-factor eating questionnaire
Engagement in health behaviors related to T2DM prevention	Surveys at baseline, 3 months, 12 months	(1) Attempting weight loss, (2) taking medication to prevent T2DM, or (3) participating in a weight-related wellness program
Mental health	Surveys at 3 and 12 months	Short form health survey (SF-12)
Weight	VA administrative data at baseline and 12 months	Weight in pounds

*T2DM* type 2 diabetes mellitus

#### Semi-structured interviews among patients with prediabetes

To gain deeper understanding of patient perceptions of a prediabetes diagnosis and brief counseling, we will conduct semi-structured telephone interviews among a maximum variation purposive sample of patients who are newly diagnosed with prediabetes and have different levels of health literacy. We will focus on health literacy because of its strong relationship to patients’ self-care behaviors, receipt of recommended preventive services, understanding of health information, and health outcomes [27]. We will conduct the interviews by phone to minimize participant burden and maximize the response rate. These participants will be invited to participate in the interview approximately 6 months after their HbA1c tests. Interviews will consist of open-ended questions to elicit feelings about receiving a prediabetes diagnosis, understanding of prediabetes and behavioral recommendations, facilitators of and barriers to behavior change, and uncertainties about having prediabetes. Each interview will be audio-recorded and transcribed verbatim, and we will stop conducting interviews when we reach thematic saturation. All interview data will be stored in password-protected files on a secure server.

### Outcomes

The primary outcome is weight change 12 months after receipt of HbA1c test results or the clinical preventive service brochure. The secondary outcomes will be changes in self-reported use of medication for prevention of T2DM, participation in a weight management program, perception of risk for T2DM, knowledge of strategies to prevent T2DM, motivation to prevent T2DM, self-efficacy to engage in behaviors to prevent T2DM, physical activity, and mental health. We will not measure progression from prediabetes to T2DM because participants in the clinical preventive service brochure arm will not have an HbA1c test as part of the study.

### Sample size calculation

Our main comparisons of interest are between outcomes for patients who receive a prediabetes diagnosis in the HbA1c test and brief counseling arm and outcomes for patients in the clinical preventive service brochure arm. Using *α* of 0.05, 56 patients who receive a prediabetes diagnosis in the HbA1c test and brief counseling arm and 56 patients in the clinical preventive service brochure arm will provide 80% power to detect a statistically significant difference between a projected mean 12-month weight change of -9.9 pounds (assuming a standard deviation (SD) of 18.0 pounds) among patients in the HbA1c test and brief counseling arm who receive a prediabetes diagnosis (comparable to mean 12-month weight change and its SD among 387 Veterans who were referred to a weight loss program in a recent clinical demonstration project at three VA medical centers [28, 29]) and a projected mean 12-month weight change of -1.4 pounds (assuming a SD of 13.5 pounds) in the clinical preventive service brochure arm (estimated from a random sample of VA patients who meet our inclusion criteria). In order to account for up to 12.5% dropout at 12 months [30] we will inflate our goal sample size for each of these groups to 63 patients.

Based on local VA data, we conservatively estimate that 25% of patients who meet our inclusion criteria will have HbA1c in the prediabetes range. Thus, to achieve our target of 63 patients with prediabetes in the HbA1c test and brief counseling arm and 63 patients in the clinical preventive service brochure arm, we will need to use a 4:1 allocation scheme to randomize 252 patients to the HbA1c test and brief counseling arm and 63 patients to the clinical preventive service brochure arm, for a total sample size of 315 patients.

### Statistical analyses

#### Quantitative analyses

For the primary outcome analysis, we will use the two-sample *t* test to evaluate the difference in weight change between patients who are found to have prediabetes in the HbA1c test and brief counseling arm and patients in the clinical preventive service brochure arm who did not receive an HbA1c test through the study. Additionally, we will compare baseline measures (e.g., age and body mass index (BMI)) in these two groups. If we find imbalance in a baseline measure (defined as *P* < 0.10 from the *F* or *X* ^2^ test), we will adjust for this measure in analyses of 12-month weight change using a mixed-effects model for longitudinal data rather than the two-sample *t* test. We will assess the extent of missing 12-month weight data and based on this assessment will consider using multiple imputation for missing data for this outcome. For secondary outcomes, we will use data from the baseline, 2-week, 3-month, and 12-month surveys to estimate mixed-effects regression models that will model mean changes between patients who are found to have prediabetes in the HbA1c test and brief counseling arm and patients in the clinical preventive service brochure arm.

#### Qualitative analyses

We will code semi-structured interview transcripts using a template analysis approach based on our conceptual model (Fig. 1) [31]. Three study team members will independently review a subset of transcripts using modified grounded theory to identify salient themes [31]. These investigators will discuss the themes, refine them, and achieve consensus on codes and definitions. Two study team members will then independently code transcripts in NVivo software. First, they will independently code the same 20% subset of the transcripts. The unweighted Cohen’s kappa statistic will then be calculated for each response and averaged to provide a single index of inter-rater reliability. Once excellent agreement is achieved, these two study team members will divide the remaining interview transcripts evenly and code these transcripts separately. The full study team will meet regularly to discuss code summaries and memos, a group consensus approach that increases the rigor of data interpretation and allows documentation of sound evidence for findings [32].

## Discussion

Nearly one in four VA patients has T2DM [33], which is a leading cause of blindness, amputations, and end-stage renal disease among Veterans and is associated with a twofold increase in annual mortality rates. Similarly, cardiovascular events resulting from T2DM and its associated risk factors lead to substantial morbidity and premature mortality among Veterans. In addition to these detrimental impacts of T2DM on the health of Veterans, these conditions contribute substantial costs within the VHA [34].

One important opportunity to limit these burdens is to facilitate patients’ engagement in efforts to prevent T2DM by informing them they have prediabetes and providing brief counseling with evidence-based recommendations. Such brief interventions have in other clinical settings effectively targeted a range of behavioral risk factors for chronic disease like cigarette smoking, alcohol misuse, and illicit drug use [35–41]. In primary care clinics, these types of interventions have also shown promise in promoting physical activity [42] and healthy dietary practices [43, 44]. A recent systematic review of brief interventions to promote physical activity and healthy dietary practices for the US Preventive Services Task Force found a positive correlation between the intensity and efficacy of these interventions, yet concluded that many higher-intensity interventions would be infeasible to implement in primary care settings [45]. Further, few lower-intensity interventions have targeted high-risk populations or leveraged behavioral science insights. Irrespective of their intensity, studies of brief interventions have also rarely reported objectively measured intermediate outcomes in addition to self-reported behaviors. As a result, the review concluded that “more trials are needed to evaluate low-intensity counseling interventions that could be more readily implemented in primary care” and that such trials should “collect and report objectively measured physiologic outcomes” [45].

In conclusion, an essential step in designing effective strategies to improve patient engagement in behavior change is to better understand their current levels of engagement in these behaviors and determine which factors influence their engagement. This RCT (SPIRIT Checklist included as Additional file 3) will generate foundational data on the effects of a prediabetes diagnosis and brief counseling on patients’ preventive behaviors and mediators of these behaviors. Our future dissemination of these results in conference proceedings and peer-reviewed publications will enable the development of novel strategies to improve patient engagement in T2DM prevention.

### Trial status

The FINDIT study began to recruit patients in December 2015.

## Additional files

Additional file 1: Standardized brief phone counseling. (DOCX 65 kb)

Additional file 2: Mailed information about HbA1c results. (DOCX 16 kb)

Additional file 3: SPIRIT checklist. (DOC 113 kb)

## Abbreviations

AAVA: Ann Arbor VA Medical Center
ADA: American Diabetes Association
BMI: Body mass index
CPRS: Computerized Patient Record System
DPP: Diabetes Prevention Program
FINDIT: Forging new paths in diabetes prevention
HbA1c: Hemoglobin A1c
MOVE: Motivating Obese Veterans Everywhere
RCT: Randomized controlled trial
SD: Standard deviation
T2DM: Type 2 diabetes mellitus
US: United States
VA: Veterans Affairs
VHA: Veterans Health Administration
