# Supplementary material for: ForgIng New paths in DIabetes PrevenTion (FINDIT): Study Protocol for a Randomized Controlled Trial
Source: Trials. 2017 Apr 8;18:167. doi: 10.1186/s13063-017-1887-6 (PMC5385070; doi:10.1186/s13063-017-1887-6)
Supplement: Supplementary file 1 — Standardized brief phone counseling. (DOCX 65 kb) [file 13063_2017_1887_MOESM1_ESM.docx]

**Additional File 1. Standardized Brief Phone Counseling**

All enclosed boxes indicate closing statements, end the call.

**Unable to reach participant**

(Be sure to never mention illness, unless you are talking directly with the participant)

1. Phone number is out of service or rings with no answer.

Please try to call directory assistance, or look in CPRS for a new phone number. Note all tries in study database.

1. Participant unavailable—answering machine

Please leave the following message:

Good morning/afternoon/evening, my name is ______ and I am calling from the VA Ann Arbor Healthcare System for Mr./Ms. _____. Please give me a call back at 1-800-753-3357. I’m usually at my desk, Monday to Friday, 8:00 am to 4:30 pm. If you cannot contact me during those times, please leave me a message with the best times to contact you and I will return your call at that time. Thank you!

1. Participant unavailable—other occupant

*Pt does not live there/deceased*

I’m sorry. We will make note in our records that this person no longer lives here/is deceased. Thank you for letting us know. Goodbye.

I’m just calling to talk to Mr./Ms. ___ about his health. I will call back later. Thank you. Goodbye.

*What is this about?*

Okay, I will try to call back then. Thank you, goodbye.

*Pt unavailable*

Is there a better time to reach him/her?

Good morning/afternoon/evening, my name is _________and I am calling from the VA Ann Arbor Healthcare System. May I please speak with Mr./Ms. ____________?

- If the potential participant is not available, **follow below**
- If you are speaking with potential participant, go to next page.

**Participant Available – Hba1c Group**

Hello, Mr./Ms. ______, I am calling from the FINDIT study. Thank you for participating in our study. You might remember that we told you when you agreed to be in our study that we would tell you the result of your Hemoglobin A1c (HbA1c) blood test. Do you have a few minutes to talk now?

*Diabetes*

*Normoglycemia*

Your HbA1C is [VALUE], which is **normal**. This means that right now you are not at risk for developing diabetes in the near future.

We have given your VA Primary Care Team your test results. Do you have any questions for me now?

If you have any questions come up, please contact us at 1-800-753-3357 or contact your Primary Care Team. We’ve also sent you a letter with this information.

We hope this information helps you achieve your health goals! Again, thank you so much for taking the time to participate in this study.

Your HbA1c is [VALUE]. Because your number is higher than 6.4% you may have **type 2 diabetes**. We have given your VA Primary Care Team these results, and they will contact you soon to schedule an appointment to talk with you about these results and the next steps. Do you have any questions for me now?

If you have any questions come up, please contact us at 1-800-753-3357 or contact your Primary Care Team. We’ve also sent you a letter with this information.

We hope this information helps you achieve your health goals! Again, thank you so much for taking the time to participate in this study.

*Prediabetes*

Your HbA1c is [VALUE]. Because this number is higher than 5.6% but less than 6.5% it means that you have **prediabetes**. In prediabetes, your blood sugar levels are higher than normal but not high enough to be called diabetes. This indicates you have an increased risk of developing diabetes soon or down the road. You are also more likely to get heart disease or have a stroke.

The good news is that you can take steps to delay or prevent getting diabetes. Here are some ways you can reduce your risk of getting diabetes.

- Lose at least 7% of your body weight
- Get at least 30 minutes of moderate physical activity (e.g., brisk walking, biking, or gardening) 5 times each week.
- A great way to achieve these goals would be to engage in a weight loss program such as the VA MOVE! program [for more information call 734-769-7100 (Ann Arbor VA) or 313- 576-1000 (Detroit VA)] or a Diabetes Prevention Program in your community (go to https://nccd.cdc.gov/DDT_DPRP/State.aspx?STATE=MI to see the list of programs in Michigan).
- Some Veterans may be able to delay or prevent type 2 diabetes by taking a medication, so you can ask your VA Primary Care Team about whether this would be right for you.

Do you have any questions for me? We have given your VA Primary Care Team your test results and just sent you a letter with this information. If any questions come up, please contact us at 1-800-753-3357 or contact your Primary Care Team. Thank you again, we hope this information helps you achieve your health goals!

**Participant Available – Brochure Group**

Hello, Mr./Ms. ______, I am calling from the FINDIT Study. Thank you for participating in our study. We just want to follow up on a brochure we sent you in the mail. Do you have a couple minutes to talk now?

We’d like to encourage you to receive all recommended screening tests and immunizations to prevent certain illnesses. We recently sent you some information on these services.

Most Veterans should receive a flu shot every year and a tetanus shot every 10 years. Do you have any questions for me?

You should talk to your health care team about what screening tests and immunizations are right for you. If any questions come up, please contact us at 1-800-753-3357 or contact your Primary Care Team. We hope this information helps you achieve your health goals! Again, thank you so much for taking the time to participate in this study.
