# Supplementary material for: ForgIng New paths in DIabetes PrevenTion (FINDIT): Study Protocol for a Randomized Controlled Trial
Source: Trials. 2017 Apr 8;18:167. doi: 10.1186/s13063-017-1887-6 (PMC5385070; doi:10.1186/s13063-017-1887-6)
Supplement: Supplementary file 2 — Mailed information about HbA1c results. (DOCX 16 kb) [file 13063_2017_1887_MOESM2_ESM.docx]

**Additional File 2. Mailed Information about HbA1c Results**

**Patients whose HbA1c is in the normal range**

Dear [PARTICIPANT NAME],

Thank you very much for participating in the FINDIT study. When you agreed to participate in this study, we mentioned we would tell you the result of your recent Hemoglobin A1C (HbA1C) blood test. This test measures how much sugar is in your blood and tells us whether you have diabetes or are at risk for getting diabetes in the near future.

Your HbA1c is [VALUE], which is **normal**. This means that right now you are not at risk for developing diabetes in the near future.

If you have any questions, please contact the FINDIT Study at 1-800-753-3357 or contact your VA Primary Care Team. We hope this information helps you achieve your health goals!

Sincerely,

Jeffrey T. Kullgren, MD, MS, MPH

Principal Investigator

VA Center for Clinical Management Research

**Notice**: This study is approved by the Ann Arbor VA Institutional Review Board.

**Patients whose HbA1c is in the prediabetes range**

Dear [PARTICIPANT NAME],

Thank you very much for participating in the FINDIT study. When you agreed to participate in this study, we mentioned we would tell you the result of your recent Hemoglobin A1C (HbA1C) blood test. This test measures how much sugar is in your blood and tells us whether you have diabetes or are at risk for getting diabetes in the near future.

Your HbA1C is [VALUE]. Because this number is greater than 5.6% but less than 6.5% it means that **you have prediabetes**. In prediabetes, your blood sugar levels are higher than normal but not high enough to be called diabetes. This indicates **you have** **an increased risk of developing diabetes soon or down the road**. You are also more likely to get heart disease or have a stroke.

The good news is that **you can take steps to delay or prevent getting diabetes**. Here are some ways you can reduce your risk of getting diabetes:

- Lose at least 7% of your body weight: <weight of 7% loss> LBS
- Get at least 30 minutes of moderate physical activity (e.g., brisk walking, biking, or gardening) 5 times each week.
- A great way to achieve these goals would be to engage in a weight loss program such as the VA MOVE! program [for more information call 734-769-7100 (Ann Arbor VA) or 313- 576-1000 (Detroit VA)] or a Diabetes Prevention Program in your community (go to https://nccd.cdc.gov/DDT_DPRP/State.aspx?STATE=MI to see the list of programs in Michigan).
- Some Veterans may be able to delay or prevent type 2 diabetes by taking a medication, so you can ask your VA Primary Care Team about whether this would be right for you.

We have given your VA Primary Care Team your test results. If you have any questions, please contact the FINDIT Study at 1-800-753-3357 or contact your Primary Care Team. We hope this information helps you achieve your health goals!

Sincerely,

Jeffrey T. Kullgren, MD, MS, MPH

Principal Investigator

VA Center for Clinical Management Research

**Notice**: This study is approved by the Ann Arbor VA Institutional Review Board.

**Patients whose HbA1c is in the T2DM range**

Dear [PARTICIPANT NAME],

Thank you very much for participating in the FINDIT study. When you agreed to participate in this study, we mentioned we would tell you the result of your recent Hemoglobin A1C (HbA1C) blood test. This test measures how much sugar is in your blood and tells us whether you have diabetes or are at risk for getting diabetes in the near future.

Your HbA1c is [VALUE]. Because your number is higher than 6.4% **you may have type 2 diabetes**. We have given your VA Primary Care Team these results, and they will contact you soon to talk with you about these results and the next steps.

If you have any questions, please contact the FINDIT Study at 1-800-753-3357 or contact your VA Primary Care Team. We hope this information helps you achieve your health goals!

Sincerely,

Jeffrey T. Kullgren, MD, MS, MPH

Principal Investigator

VA Center for Clinical Management Research

**Notice**: This study is approved by the Ann Arbor VA Institutional Review Board.
